# Effects of NGF, NT-3 and GDNF family members on neurite outgrowth and migration from pelvic ganglia from embryonic and newborn mice

**DOI:** 10.1186/1471-213X-8-73

**Published:** 2008-07-25

**Authors:** Ashley L Stewart, Richard B Anderson, Kazuto Kobayashi, Heather M Young

**Affiliations:** 1 Department of Anatomy and Cell Biology, University of Melbourne, 3010, Australia; 2 Department of Molecular Genetics, Institute of Biomedical Sciences, Fukushima Medical University, Fukushima, 960-1295, Japan

## Abstract

**Background:**

Pelvic ganglia are derived from the sacral neural crest and contain both sympathetic and parasympathetic neurons. Various members of the neurotrophin and GDNF families of neurotrophic factors have been shown to play important roles in the development of a variety of peripheral sympathetic and parasympathetic neurons; however, to date, the role of these factors in the development of pelvic ganglia has been limited to postnatal and older ages. We examined the effects of NGF, NT-3, GDNF, neurturin and artemin on cell migration and neurite outgrowth from explants of the pelvic ganglia from embryonic and newborn mice grown on collagen gels, and correlated the responses with the immunohistochemical localization of the relevant receptors in fixed tissue.

**Results:**

Cell migration assays showed that GDNF strongly stimulated migration of tyrosine hydroxylase (TH) cells of pelvic ganglia from E11.5, E14.5 and P0 mice. Other factors also promoted TH cell migration, although to a lesser extent and only at discrete developmental stages. The cells and neurites of the pelvic ganglia were responsive to each of the GDNF family ligands – GDNF, neurturin and artemin – from E11.5 onwards. In contrast, NGF and NT-3 did not elicit a significant neurite outgrowth effect until E14.5 onwards. Artemin and NGF promoted significant outgrowth of sympathetic (TH+) neurites only, whereas neurturin affected primarily parasympathetic (TH-negative) neurite outgrowth, and GDNF and NT-3 enhanced both sympathetic and parasympathetic neurite outgrowth. In comparison, collagen gel assays using gut explants from E11.5 and E14.5 mice showed neurite outgrowth only in response to GDNF at E11.5 and to neurturin only in E14.5 mice.

**Conclusion:**

Our data show that there are both age-dependent and neuron type-dependent differences in the responsiveness of embryonic and neo-natal pelvic ganglion neurons to growth factors.

## Background

The pelvic ganglia provide the majority of autonomic innervation to the urogenital organs and part of the extrinsic innervation of the lower bowel [1-3]. In humans, the plexus is extensive and is frequently injured during pelvic surgical procedures [4-7]. The development of regenerative therapies may be facilitated by improved knowledge of the processes that occur during the normal development of the pelvic neuronal circuits. In mice and rats, the structure of the pelvic ganglia is simpler than in humans, and consists of paired, morphologically discrete, major pelvic ganglia [8]. Hence, developing rodent pelvic ganglia are an accessible system in which to study developmental processes involved in formation of pelvic autonomic circuits [9].

Unlike other autonomic ganglia, the pelvic ganglia are comprised of a mixture of sympathetic and parasympathetic post-ganglionic neurons [1,2,10]. Both sympathetic and parasympathetic post-ganglionic neurons of pelvic ganglia are derived from the sacral neural crest [11-14]. In embryonic mice, sacral neural crest cells migrate ventrally and coalesce into aggregates between the distal hindgut and the urogenital sinus [11,15]. Some sacral neural crest cells contribute neurons and glial cells to distal regions of the gut [12]; these cells reside transiently within the pelvic plexus primordia for around 3 days before entering the hindgut [11,15-17].

The factors that regulate the migration of sacral neural crest cells and the axonal projections of the developing pelvic ganglia remain largely unknown. In contrast, factors regulating migration and axon extension in other components of the autonomic nervous system including the enteric nervous system, sympathetic ganglia, and cranial parasympathetic ganglia have been well studied [18-28]. As neural crest from different axial levels possess different developmental capabilities, and *in vitro*, differ in their response to some factors [29], the mechanisms regulating cell differentiation in the sacral neural crest-derived sympathetic and parasympathetic neurons may be different from those in more rostral sympathetic and parasympathetic ganglia.

Axon extension and neural migration in the peripheral nervous system is influenced by numerous neurotrophic factors. The best characterized group is the neurotrophin family that consists of four members – nerve growth factor (NGF), neurotrophin-3 (NT-3), brain-derived neurotrophic factor (BDNF), and neurotrophin-4 (NT-4). There are three different tyrosine kinase receptors that mediate the effects of neurotrophins – TrkA, TrkB and TrkC [30]. NGF binds and activates the TrkA receptor, BDNF and NT-4 both signal through TrkB, and NT-3 activates TrkC. While NGF, BDNF and NT-4 show very little receptor promiscuity, NT-3 can under some circumstances also interact with the TrkA and TrkB receptors [31].

The GDNF family ligands (GFLs) – glial cell-line-derived neurotrophic factor (GDNF), neurturin (NRTN) and artemin (ART) are important neurotrophic factors for many types of neurons including central motor, dopamine, and noradrenaline neurons as well as for sub-populations of peripheral autonomic and sensory neurons [32-42]. All GFLs signal through a receptor complex composed of a common signaling subunit, the Ret receptor tyrosine kinase. The ligand specificity is determined by a co-receptor subunit – GDNF, neurturin and artemin bind preferentially to GFRα1, GFRα2 and GFRα3 respectively [43]. GFRα receptors are usually bound to the plasma membrane, but can also be cleaved to produce soluble forms [44].

A number of studies have examined the expression of neurotrophic factor receptors by pelvic ganglion neurons and ligand expression in their targets in post-natal and adult animals. A variety of neurotrophic factors including neurotrophins and members of the GDNF family are expressed in tissues innervated by pelvic neurons including the colon, penis, vas deferens and bladder [45-51]. Furthermore, there are changes in pelvic neurons and their innervation of targets in neurotrophic factor ligand- or receptor-deficient mice; in mice lacking neurturin, or its receptor GFRα2, there are defects in the parasympathetic innervation of some of the reproductive organs [3,50,52]. *In vitro *studies have shown that neurturin stimulates soma growth and promotes neurite extension of dissociated parasympathetic neurons from adult male rats [53]. However, these studies demonstrate that neurturin is only essential in some pelvic target organs.

While the role of neurotrophins and neurturin in the adult [49-58], and more recently, postnatal [3] pelvic plexus has been examined, there is relatively little known about the role of different neurotrophic factors in the pre-natal development of the pelvic ganglia. In mice, GDNF and neurturin are expressed by a variety of pelvic tissues at mid-embryonic ages including the colon (from E10), genital tubercle (from E10) and urogenital sinus (from E14), ovary (from E14) and testicle (from E14) [59], but the expression of neurotrophins and artemin does not appear to have been examined pre-natally.

In the present study, we report the effects of neurotrophin and GFL signalling on cell migration and neurite outgrowth of parasympathetic and sympathetic neurons in pelvic ganglia from embryonic and newborn mice and correlate the responses with the expression of the corresponding receptors. Our data show that GDNF family members have actions from early stages (E11.5) of pelvic plexus development, while neurotrophins do not exert an influence until later. As in older animals, sympathetic and parasympathetic neurons in pelvic ganglia from embryonic and newborn mice show differences in their responses to a number of neurotrophic factors.

## Methods

### Animals

Embryonic C57/Bl6 mice were used for all explant culture experiments. Mice in which the expression of GFP was driven by the rat tyrosine hydroxylase (TH) promoter [60] were used for receptor localisation studies. TH-GFP mice were on a C57/BL6 background. The genotype of adult TH-GFP mice was determined by PCR using the primers and conditions previously reported [60]. Male mice heterozygous for the TH-GFP allele (*TH*^*GFP*/+ ^mice) were mated to wild-type (C57/BL6) females. To distinguish *TH*^*GFP*/+ ^from *wild-type *embryos, the sympathetic chain of each embryo was examined under a fluorescence microscope to determine if GFP+ cells were present; only *TH*^*GFP*/+ ^embryos were used. Timed pregnant mice were killed by cervical dislocation. The morning on which a copulatory plug was observed was designated E0.5. All procedures were approved by the University of Melbourne Animal Experimentation Ethics Committee.

### Pelvic plexus explants grown on collagen gels

The location of pelvic plexus primordium in E11.5 and E14.5 mice has been described previously [11,15]. In E11.5 mice, the plexus primordia are dorso-lateral to the distal hindgut, and in E14.5 mice, they are ventro-lateral to the distal hindgut. In P0 male mice, discrete ganglia are located on the dorsal surface of the prostate gland, and can be recognized macroscopically [11,15]. Connective tissue containing pelvic plexus primordia (E11.5, E14.5) or ganglia (P0) were dissected from E11.5, E14.5 and P0 mice and placed in culture medium (DMEM containing 10% foetal bovine serum, 2 mM glutamine, 0.075% sodium bicarbonate and penicillin/streptomycin sulfate solution), and were grown on collagen gels as described previously [61,62]. Briefly, acidic collagen solution (4 mg/ml; Upstate, CA, USA) was restored to normal osmolality with 5 × Dulbecco's modified Eagle's medium (DMEM) and normal pH with 200 mM NaOH, on ice. This solution was diluted to 1 mg/ml with culture medium. Growth factor was added to the collagen solution prior to gelling to give a final concentration of 100 ng/ml GDNF, artemin, neurturin or NGF; or 40 ng/ml NT-3 (Peprotech, NJ, USA). These concentrations are based on previous studies that have examined the responses of sympathetic and enteric neurons from embryonic and post-natal mice *in vitro *[61,63,64]. Each explant contained an entire ganglion. Transverse slices from E11.5 midgut and E14.5 hindgut, as well as dorsal root ganglia from E11.5, E14.5 or P0 mice, were also grown on control and growth-factor impregnated gels.

### Immunohistochemical analysis of cryosections

The pelvic region of *TH*^*GFP*/+ ^embryos was fixed overnight in 4% paraformaldehyde in 0.1 M phosphate buffer. Cryosections of 12 μm thickness were cut transverse to the neural tube and then processed for immunohistochemistry using antisera for Hu, GFP and either GFRα1, GFRα2, GFRα3, TrkA or TrkC, as shown in Table 1. Characterizations of the GFRα antibodies used have been recently described [65]. Moreover, the GFRα1 antibody gives an identical pattern of staining to EGFP expression in *Gfrα1*^*EGFP *^mice [66]. Previous studies have shown that the TrkA antibody does not cross react with TrkB or TrkC [67]. The immunogen sequence used to raise the TrkC antibody was chosen to avoid cross reactivity with TrkA and TrkB (manufacturers' information). Sections exposed to each of the secondary antisera only showed no staining.

**Table 1:** Primary and secondary antibodies

**Primary antibodies**	**Source**	**Secondary antibodies**	**Source**
Sheep anti-tyrosine hydroxylase	Chemicon, Temecula, CA, USA	anti sheep-FITC	Jackson Immunoresearch, West Grove, PA, USA
Mouse anti-Tuj-1	Covance Research, Berkeley, CA, USA	anti mouse-594	Molecular Probes
Human anti-Hu	Dr Miles Epstein; [99]	anti human-Texas Red	Jackson Immunoresearch
Goat anti-GFP	Rockland Immunochemicals, Gilbertsville, PA, USA	anti sheep-FITC	Jackson Immunoresearch
Rabbit anti-GFP	Molecular Probes, Eugene, OR, USA	anti rabbit-FITC	Jackson Immunoresearch
Goat anti-GFRα1, GFRα2 or GFRα3	R&D Systems, Minneapolis, MN, USA	anti sheep-CY5	Molecular Probes
Rabbit anti-TrkA	Dr Louis Reichardt; [67]	anti rabbit-CY5	Molecular Probes
Rabbit anti-TrkC	Chemicon	anti rabbit-CY5	Molecular Probes
Rabbit anti-B-FABP	Dr Thomas Müller; [69]	anti rabbit-FITC	Jackson Immunoresearch

### Quantification of process outgrowth and cell migration

Explants grown on collagen gels were imaged under a Leica MZ16F fluorescence stereomicroscope. Explants were immunostained using antibodies to Tuj-1 and TH. Tuj-1 immunostaining will identify all neurites and TH immunostaining will identify the cell bodies and neurites of most sympathetic neurons (see Discussion). To quantify cell and neurite outgrowth from collagen gel explants, a feathered, Otsu-algorithm thresholded mask was applied to each channel, excluding the explant, to eliminate noise from regions without outgrowth (ImageJ and Adobe Photoshop, CA, USA) as described previously [62]. Three thresholded images were then produced for quantification corresponding to Tuj-1 fibres, TH-fibres and TH-cells. The total area of stained pixels was obtained, as an estimate of the total number of cells/fibres outside the explant. The quantification of Tuj-1 fibres and TH-fibres provided information about the area occupied by stained fibres outside the explant, but do not provide any information about the length of individual fibres or intensity of immunostaining. To estimate neurite outgrowth from parasympathetic neurons in the explants, the area of TH immunostaining was subtracted from the area of Tuj-1 immunostaining. The quantification of TH+ cell bodies separately from TH-fibres was possible due to TH+ cell bodies possessing an increased intensity of immunofluorescence compared to TH+ fibres, allowing their differential selection by thresholding. The quantification of TH+ cells outside of the explants provided information about the area occupied by TH+ cell bodies outside the explants, but do not provide any information about the distance of the TH+ cell bodies from the explants. TH+ cells had to be greater than one cell body diameter away from the explant to be considered outside of the explant. Measurements were made using ImageJ (NIH, USA). Results are reported as mean ± standard error of the mean. Statistical analyses were performed using one way ANOVAs followed by Dunnett's *post-hoc *tests. A *p *value of < 0.05 was deemed significant. The data for each age and each factor are based on analysis of a minimum of 3 experiments and a minimum of 7 explants.

## Results

### Response of pelvic plexus neurons to neurotrophic factors

**Figure 1:**
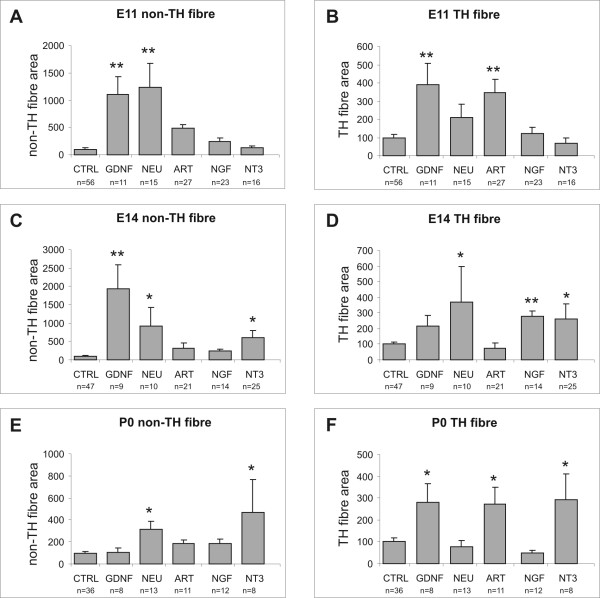
**Quantification of the effects of glial cell line-derived neurotrophic factor (GDNF), neurturin, artemin, NGF and NT3 on non-TH+ (area of Tuj-1 immunostaining minus area of TH immunostaining; A, C, E) and area of TH+ (B, D, F) fibre outgrowth from pelvic plexus explants taken at embryonic day (E) 11.5 (A-B), embryonic day (E) 14.5 (C-D) and postnatal day (P) 0 (E-F) and cultured for 4 days.** The data (mean ± SEM) show the immunostained area as a percentage of mean immunostained area of control explants at each developmental stage. Significant responses are indicated with asterisks (one way ANOVA followed by Dunnett's *post-hoc *test; * – *p *< 0.05, ** – *p *< 0.01). "n"s refer to the number of explants analysed.

**Figure 2:**
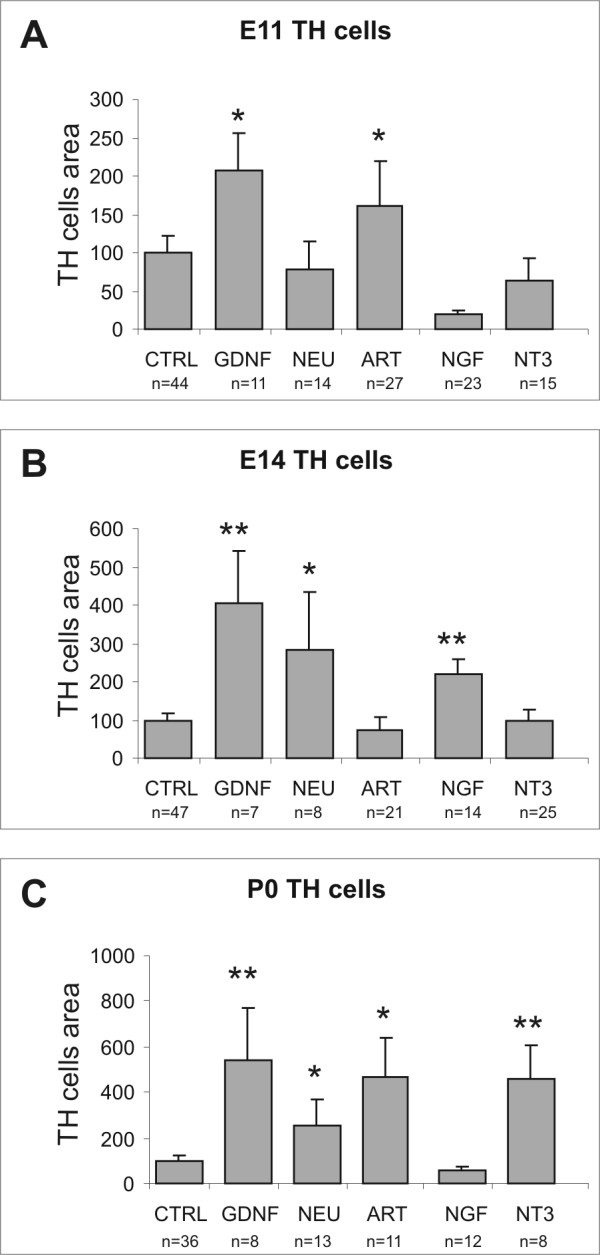
**Quantification of the effects of glial cell line-derived neurotrophic factor (GDNF), neurturin, artemin, NGF and NT3 on area occupied by TH+ cells outside the explants, from pelvic plexus explants taken at embryonic day (E) 11.5 (A), embryonic day (E) 14.5 (B) and postnatal day (P) 0 (C) and cultured for 4 days.** The data (mean ± SEM) show the immunostained area as a percentage of mean immunostained area of control explants at each developmental stage. Significant responses are indicated with asterisks (one way ANOVA followed by Dunnett's *post-hoc *test; * – *p *< 0.05, ** – *p *< 0.01). "n"s refer to the number of explants analysed.

Explants of E11.5 or E14.5 pelvic plexus primordium or P0 pelvic ganglia were grown on collagen gels, with or without 40 ng/ml NT-3, or 100 ng/ml GDNF, artemin, neurturin or NGF. After 4 days in culture, the explants were immunostained for Tuj-1 and TH; Tuj-1 immunostaining will identify all neurites and TH immunostaining will identify the cell bodies and neurites of sympathetic neurons. To estimate neurite outgrowth from parasympathetic neurons in the explants, the TH staining was subtracted from the Tuj-1 staining, which gives the area occupied by TH-negative neurites. In addition, the migration of TH (sympathetic) cell bodies was quantified. The data are summarized in Figures 1 and 2.

#### Control explants

**Figure 3:**
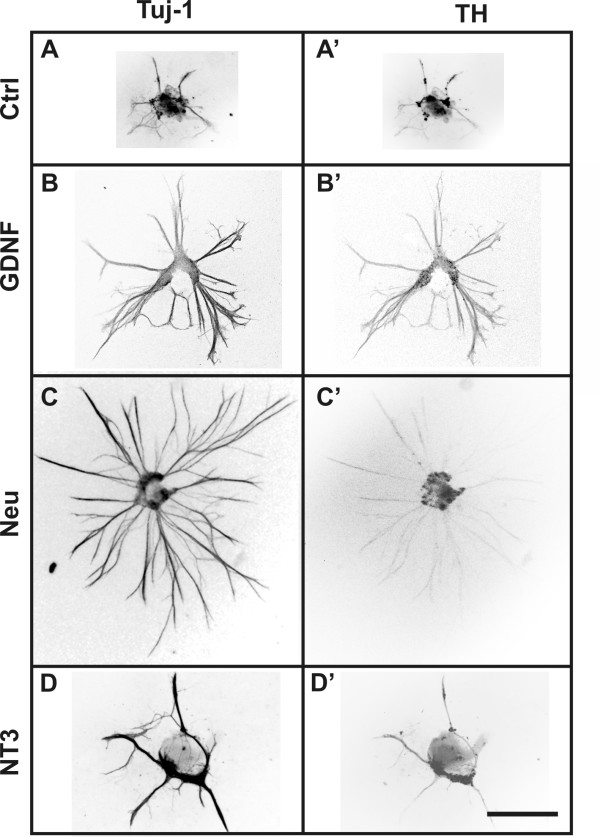
**Inverted fluorescence micrographs of embryonic day (E) 11.5 pelvic plexus explants grown on collagen gels impregnated with various neurotrophic factors (A-D').** After 4 days, the explants were processed for Tuj-1 (A, B, C, D) and TH (A', B', C', D') imunohistochemistry. There was limited Tuj-1+ (A) and TH+ (A') fibre outgrowth from control gels. GDNF-impregnated gels produced significant Tuj-1+ (B) and TH+ (B') fibre outgrowth; whereas Neurturin treated gels only produced significant Tuj-1+ fibre outgrowth (C), with little TH+-fibre outgrowth (C'). The level of outgrowth from NT-3-impregnated gels was not significant (D-D'). Scale bar = 500 μm

**Figure 4:**
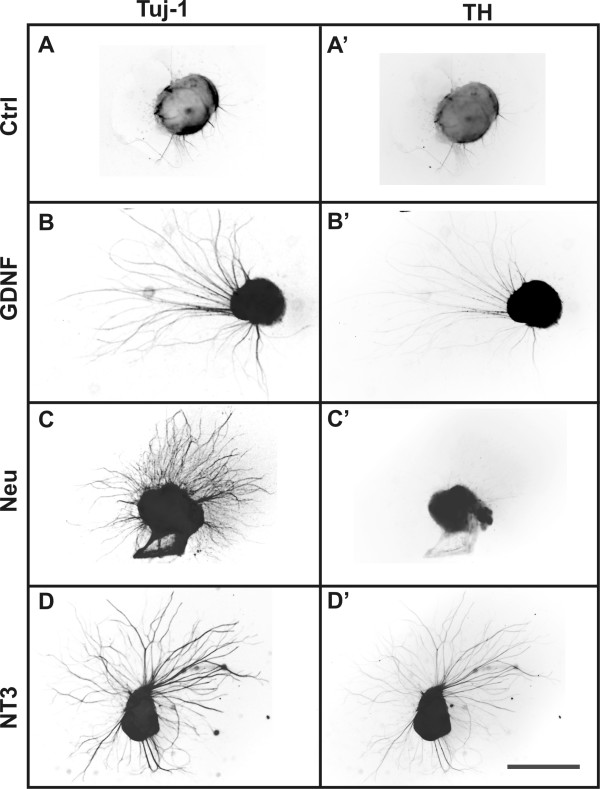
**Inverted fluorescence micrographs of postnatal day (P) 0 pelvic plexus explants grown on collagen gels impregnated with various neurotrophic factors (A-D').** After 4 days, the explants were processed for Tuj-1 (A, B, C, D) and TH (A', B', C', D') imunohistochemistry. There was limited Tuj-1+ (A) and TH+ (A') fibre outgrowth from control gels. GDNF-impregnated gels produced significant Tuj-1+ (B) fibre outgrowth, almost all of which was TH+ (B') fibre outgrowth. Neurturin treated gels also stimulated significant Tuj-1+ fibre outgrowth (C), almost all of which was not TH+-fibre outgrowth (C'). The level of Tuj-1+ (D) and TH+ (D') fibre outgrowth from NT-3-impregnated gels was significant. Scale bar = 500 μm

Explants grown on control gels (devoid of any neurotrophic factor) did exhibit some basal neurite outgrowth (eg. Figs. 3A-A'; 4A-A') and TH cell migration. Explants of E11.5, E14.5 or P0 DRG grown on collagen gels also exhibited some neurite outgrowth in the absence of neurotrophic factors (data not shown). In contrast, there was almost no cell migration or neurite extension from explants of E11.5 midgut or E14.5 hindgut in the absence of neurotrophic factor (see below).

#### E11.5 explants

In explants of pelvic plexus primordium from E11.5 mice, parasympathetic fibre outgrowth was significantly increased by both GDNF and neurturin (12-fold and 13-fold change compared to control explants, respectively; *p *< 0.01), whereas artemin did not elicit a significant response (Fig. 1A). Neither NGF nor NT-3 affected the parasympathetic fibre response (Fig. 1A). We observed a significant increase in outgrowth of TH fibres in the presence of both GDNF and artemin (4-fold and 3.5-fold, respectively; *p *< 0.01; Figs. 1B, 3B' and 3C'), but not in the presence of neurturin, NGF or NT-3 (Figs. 1B and 3D'). Significant increases in TH+ cell migration were only observed in the presence of GDNF and artemin (2-fold and 1.5-fold, respectively; *p *< 0.05; Fig. 2A).

#### E14.5 explants

Parasympathetic fibre outgrowth was significantly increased by GDNF (17-fold, *p *< 0.01) and neurturin (9-fold, *p *< 0.05; Fig. 1C). Unlike E11.5 explants, NT-3 induced a significant parasympathetic outgrowth from E14.5 explants (6-fold, *p *< 0.05; Fig. 1C). NGF and artemin did not promote parasympathetic outgrowth from E14.5 explants. GDNF and artemin no longer stimulated a significant TH+ fibre outgrowth, whereas increased TH+ fibre outgrowth was observed in response to neurturin, NGF, and NT-3 (3.5-fold, 3-fold, and 3-fold, respectively; *p *< 0.05; Fig. 1D). In the presence of NT-3, neurites appeared to be more fasciculated than in control explants or explants grown in the presence of the other growth factors. In E14.5 explants, GDNF continued to elicit TH+ cell migration (4-fold, *p *< 0.01; Fig. 2B). Neurturin and NGF were also able to elicit significant TH+ cell migration (3-fold, *p *< 0.05; and 2-fold, *p *< 0.01; respectively; Fig. 2B). However, unlike E11.5 explants, no significant TH+ cell migration was observed in response to artemin, and, despite inducing TH+ fibre outgrowth at this age, NT-3 did not promote TH+ cell migration (Fig. 2B).

#### P0 explants

A number of differences in the response of pelvic ganglia explants to the various growth factors were observed in P0 explants compared to E11.5 and E14.5 explants. Parasympathetic fibre outgrowth continued to be stimulated in response to both neurturin and NT-3 (8.5-fold and 12-fold, respectively; *p *< 0.05; Fig. 1E). However, GDNF no longer produced a parasympathetic fibre outgrowth response (Fig. 1E). NT-3 stimulated TH+ fibre outgrowth (3-fold; *p *< 0.05; Figs. 1F and 4D'); and, similar to their effect on E11.5 but not E14.5 explants, GDNF and artemin elicited significant TH+ fibre outgrowth responses from P0 explants (3-fold and 2.5-fold, respectively; *p *< 0.05; Figs. 1F and 4B'). All of the growth factors except NGF induced significant increases in TH+ cell migration (GDNF: 5.5-fold, NT-3: 4.5-fold, *p *< 0.01; neurturin: 2.5-fold, artemin: 4.5-fold, *p *< 0.05; Fig. 2C).

### Response of E11.5 midgut and E14.5 hindgut explants to NT-3, NGF and GDNF family members

**Figure 5:**
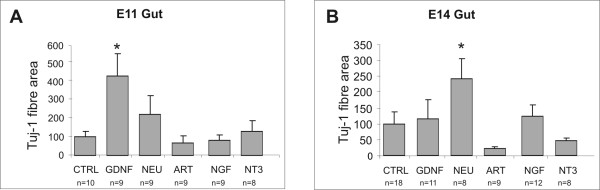
**Quantification of the effects of glial cell line-derived neurotrophic factor (GDNF), neurturin, artemin, NGF and NT3 on area of Tuj-1+ fibre outgrowth, from slices of embryonic day (E) 11.5 midgut (A), or embryonic day (E) 14.5 hindgut (B), cultured for 4 days.** The data (mean ± SEM) show the area of immunostaining as a percentage relative to the mean immunostained area of Tuj-1+ fibres obtained from control explants at each developmental stage. Significant responses to GDNF at E11.5 (A), and neurturin at E14.5 (B) are indicated with an asterisk (one way ANOVA followed by Dunnett's *post-hoc *test; * – *p *< 0.05). "n"s refer to the number of explants analysed.

Transverse slices of E11.5 midgut, or E14. 5 hindgut were grown on collagen gels, with or without 40 ng/ml NT-3, or 100 ng/ml GDNF, artemin, neurturin or NGF. The data are summarized in Figure 5.

As previously shown [64], E11.5 and E14.5 gut explants grown on control gels (devoid of any neurotrophic factor) or in the presence of artemin, exhibit little or no neurite outgrowth (Fig. 5A); which is in contrast to pelvic plexus explants, which show some basal level of neurite extension in the absence of added neurotrophic factor. We observed a significant neurite outgrowth response to GDNF from E11.5 midgut slices (GDNF: 4-fold, *p *< 0.05; Fig. 5A). At E14.5, only neurturin induced significant neurite outgrowth (2.5-fold, *p *< 0.05) and there was little or no neurite outgrowth in response to GDNF (Fig. 5B). Although TrkC is expressed by a sub-population of neural crest derived cells in the gut and NT-3 has effects on differentiation [68], the effects of NGF and NT-3 on neurite outgrowth from gut slices has not been previously examined. Neither NGF nor NT-3 elicited a significant increase in total (Tuj-1+) neurite outgrowth at either E11.5 or E14.5 (Fig. 5A and 5B, respectively).

### Expression of receptors for NGF, NT-3 and GDNF family members during development of pelvic ganglia

Immunohistochemical localisation of TrkA, TrkC, GFRα1, GFRα2 and GFRα3 was performed on cryosections through the pelvic region of E11.5, E14.5 and P0 TH-GFP mice. The overlap in expression of the receptors with Hu, a pan-neuronal marker, and GFP (TH) was also examined. The results are summarized in Table 2.

**Table 2:** Expression of receptors for NGF, NT-3 and GDNF family members during development of pelvic ganglia

	**TrkA**		**TrkC**		**GFRα1**		**GFRα2**		**GFRα3**	
	
	**Hu+/TH+**	**Hu+/TH-**	**Hu+/TH+**	**Hu+/TH-**	**Hu+/TH+**	**Hu+/TH-**	**Hu+/TH+**	**Hu+/TH-**	**Hu+/TH+**	**Hu+/TH-**
**E11.5**	-	-	+	-	+	+	+	+	-	-
**E14.5**	+	+	+	-	+	+	+	+	+	+
**P0**	+	-	+	+	-	+	+	+	Neuropil only	-

- no detectable immunoreactivity; + immunstaining observed

#### E11.5

**Figure 6:**
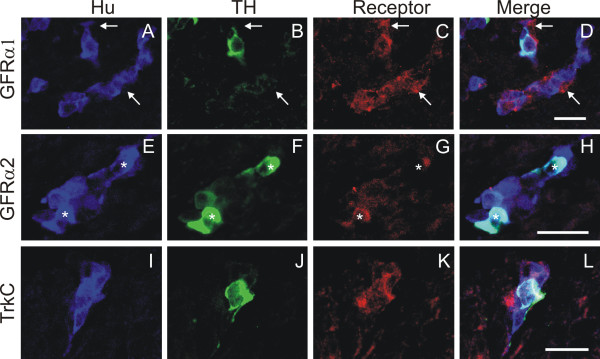
**Confocal images of transverse sections through the pelvic region at the level of the pelvic plexus primordium of E11.5 TH-GFP mice processed for Hu (blue), GFP (TH, green) and either GFRα1 (red) (A-D), GFRα2 (red) (E-H) or TrkC (red) (I-L) immunohistochemistry.** Chains of Hu+ cells were present in the pelvic plexus primordium (A, E, I). A sub-population of the Hu+ cells was also GFP+ (TH+) (A-B, E-F, I-J). GFRα1 immunoreactivity was present on Hu+/TH-negative neurons, as well as some TH+/Hu+ neurons and some cells in the clusters that were TH-/Hu- (*arrows*) (A-D). Weak GFRα2 immunostaining was found on all Hu+ cells in the pelvic plexus primordium; however, strong GFRα2 immunoreactivity was found on many of the Hu+/TH+ cells (*asterisks*), whereas GFRα2 immunorectivity was weak on the Hu+/TH-negative sub-population (E-H). TrkC immunostaining was mainly observed on the Hu+/TH+ sub-population (I-L). Scale bars = 20 μm.

In E11.5 mice, the pelvic plexus primordia were present bilaterally in small, dispersed clusters located dorsolateral to the distal hindgut, as previously reported [15]. Many of these cells were Hu+, and a sub-population of the Hu+ cells was also TH+ (Fig. 6). Most Hu+/TH- neurons were GFRα1+ (Fig. 6A–D). In addition, some Hu+/TH+ neurons and some Hu-/TH- cells within the cell clusters were also GFRα1+ (Fig. 6A–D). Hu-/TH- cells are likely to be neural progenitors. GFRα2 was only expressed by Hu+ cells; the intensity of GFRα2 staining appeared higher on TH+ cells, while TH-negative cells exhibited relatively weak GFRα2 immunoreactivity (Fig. 6E–H). Most Hu+/TH+ cells were TrkC+ (Fig. 6I–L). Cells within the pelvic plexus primordia did not exhibit any immunostaining for TrkA or GFRα3 (data not shown). However, TrkA staining was present on cells within the sympathetic ganglia and DRG (data not shown).

#### E14.5

The pelvic plexus primordium is now located ventrolateral to the gut and dorsolateral to the urogenital sinus [15]. A sub-population of the Hu+ cells was also TH+. TrkA staining was first evident at this stage and was present primarily on the Hu+/TH+ cells, although some Hu+/TH- cells also were TrkA+ (data not shown). Similarly, Hu+/TH+ cells were found to show strong TrkC immunostaining, but no TrkC staining was observed on the Hu+/TH- sub-population of cells (data not shown). GFRα2 immunostaining was also present primarily on the Hu+/TH+ sub-population of cells, with most of the Hu+/TH- population only weakly GFRα2+. By contrast, GFRα1 was localised primarily to a subgroup of the Hu+/TH- cells, with only weak GFRα1 staining found on the Hu+/TH+ sub-population (data not shown). GFRα3 staining was uniformly weak across all Hu+ cells (data not shown).

#### P0

**Figure 7:**
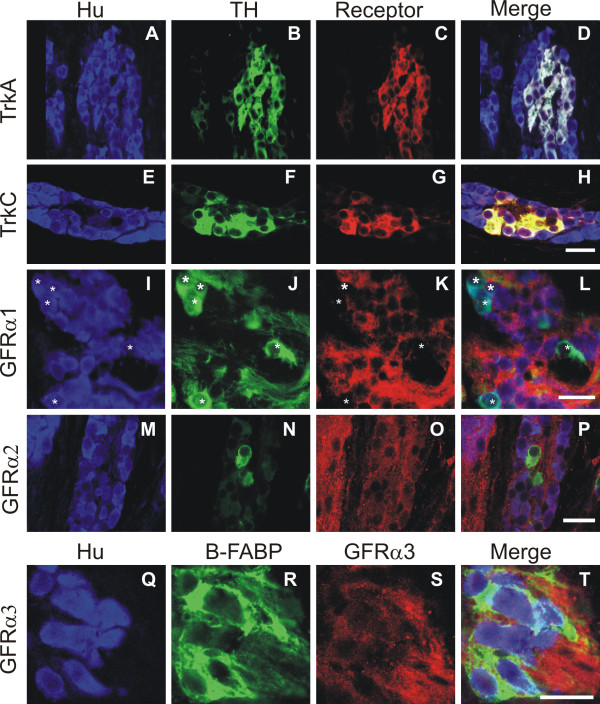
**Confocal images of transverse sections through the pelvic ganglia from postnatal (P) 0 day mice processed for Hu (blue), TH (green) and either TrkA (red) (A-D), TrkC (red) (E-H), GFRa1 (red) (I-L), or GFRa2 (red) (M-P); or for Hu (blue), B-FABP (green) and GFRa3 (red) (Q-T) immunohistochemistry.** A sub-population of cells immuno-positive for the pan-neuronal marker Hu are also TH+ (A-B, E-F, I-J, O-N, Q-R). These Hu+/TH+ cells are distributed throughout the ganglia, sometimes forming discrete clusters (B, F), othertimes more dispersed (J, N). Strong levels of TrkA (A-D), and TrkC (E-H) immunostaining were observed on the Hu+/TH+ sub-population of cells, although Hu+/TH-negative cells were also weakly TrkC immuno-positive (E-H). In contrast, GFRα1 immunostaining was observed only on the Hu+/TH-negative population of cells, and not on the Hu+/TH+ cells (*asterisks*). GFRα2 immunostaining was found to be ubiquitously expressed on all Hu+ cells throughout the pelvic ganglia (M-P). GFRα3 immunostaining was found to be on the neuropil only as it did not co-localise with either the pan-neuronal marker Hu or the glial marker B-FABP (Q-T). Scale bars = 20 μm.

In P0 male mice, the pelvic ganglia are located near to the dorsal surface of the prostate, closely associated with the urogenital organs and hindgut [9]. A sub-population of Hu+ neurons was also TH+ (Fig. 7A–B, 7E–F, 7I–J, 7M–N). The proportion of Hu+/TH- to Hu+/TH+ neurons varied throughout the ganglia with some regions almost exclusively Hu+/TH- or Hu+/TH+. TrkA staining was found exclusively on the Hu+/TH+ sub-population of neurons (Fig. 7A–D). The Hu+/TH+ sub-population of neurons also showed a high intensity of TrkC immunostaining, while Hu+/TH- neurons were only weakly TrkC+ (Fig. 7E–H). In contrast, GFRα1 was present predominantly on the Hu+/TH- cells (Fig. 7I–L). All Hu+ cells within the ganglia were weakly GFRα2+ (Fig. 7M–P), while nerve fibres were strongly GFRα2+. GFRα3 immunostaining was found only in Hu- regions of the ganglia. Double staining with the glial precursor marker B-FABP [69,70] also revealed no overlap between B-FABP and GFRα3 (Fig. 7Q–T). GFRα3 immunostaining therefore appeared to be associated with the neuropil.

## Discussion

We have used collagen gel assays to investigate the role of the GFLs and neurotrophins in pelvic plexus development in embryonic and newborn mice. Our data show that neurons in the pelvic plexus primordium of E11.5 mice show neurite outgrowth and cell migration responses to GFLs. By contrast, an effect of the neurotrophins was not seen until E14.5. Most of the sympathetic neurons in pelvic ganglia are TH+ [2]. To estimate neurite outgrowth from parasympathetic neurons in the explants in the current study, the TH staining was subtracted from the Tuj-1 staining. A caveat of these methods is that definitions of "sympathetic" and "parasympathetic" should be based on neuroanatomical inputs rather than chemistry [2]. For example, there are some TH-negative sympathetic neurons in pelvic ganglia [2] and thus a small number of TH-negative neurites analysed in the current study are likely to arise from sympathetic neurons. Our data show that both the sympathetic (TH+) and parasympathetic (TH-negative) sub-populations of neurons are responsive to GDNF and NT-3, sympathetic neurons are primarily responsive to artemin and NGF, while parasympathetic neurons are mainly responsive to neurturin.

### Effects of neurotrophic factors on pelvic plexus cell migration

Of the receptor components studied, only GFRα1 was found to be expressed on Hu-negative sacral crest-derived pelvic plexus cells at E11.5. These cells are probably cells of the neural crest-derived progenitor pool, but may also be glial precursors. As all other growth factor receptors were only found on Hu+ cells, it appears that only differentiated neurons are responsive to NGF, NT-3, artemin and neurturin. GDNF-GFRα1 signaling could therefore potentially regulate the very early stages of pelvic ganglia formation, with possible actions on guidance, differentiation, proliferation or survival of the undifferentiated sacral neural crest cells.

GDNF exhibited the most potent chemotactic action on cell migration of pelvic plexus neurons of all the factors examined; GDNF stimulated migration of TH+ cells from pelvic plexus explants at E11.5, E14.5 and P0 mice. The finding that GDNF produces significant TH+ cell migration at all stages is surprising, as TH+ cells did not express detectable levels of GFRα1. There are three possible explanations: first, the migration-promoting action of GDNF is not through its canonical receptor GFRα1. GFRα2 is expressed by TH+ cells, and it has been reported that, *in vitro*, GDNF can actively signal through binding to GFRα2 [71]. However, neurturin, which most strongly activates the GFRα2 receptor, does not elicit a significant cell migration response at E11.5, and thus this possibility appears unlikely. Second, GDNF may act on the Hu-/TH- and/or Hu+/TH- sub-populations of cells, which do express GFRα1; these cells migrate in response to GDNF and then subsequently up-regulate TH expression. Finally, the number of GFRα1 receptors required to mediate a biological response might be below the detection threshold for immunohistochemical detection.

The timing of effect of the other growth factors on TH+ cell migration also gives the latter explanation further credence, as in each case the stages at which TH+ cell migration occurred correlated with the expression of the relevant receptors on the Hu+/TH- sub-population, but does not correlate with the expression of the relevant receptors on the Hu+/TH+ sub-population. Further studies are required to examine the migratory ability of Hu+/TH+, Hu+/TH- and Hu-/TH- cells.

### Responses to neurotrophins

NGF is widely expressed within the target pelvic organs of neurons of the pelvic ganglia [49,72-78]; and the neurotrophin receptors trkA and p75 are expressed by adult noradrenergic pelvic ganglion neurons [49,54], as would be expected for NGF-sensitive neurons [79,80]. Furthermore, in mice in which both the NGF and Bax genes were knocked-out to permit the role of NGF in target innervation to be determined separately from the role of NGF in survival, there were impairments in the density of sympathetic nerves in the urinary bladder, ureter and gonads, demonstrating a role for NGF in axon pathfinding and/or axon growth and branching in these targets [81]. Thus, it has been assumed that many or all pelvic noradrenergic neurons are potentially affected by endogenous NGF. However, we found NGF to be the neurotrophic factor least effective at eliciting responses from the pelvic ganglia in embryonic and newborn mice, resulting in a small but significant increase in TH+ cell and fibre outgrowth only in E14.5 mice. An earlier study using Remak's ganglia in chick, which are also derived from the sacral neural crest and are comprised of both sympathetic and parasympathetic neurons – also found a weak response to NGF throughout embryonic development with a limited period (E8–E12) of fibre outgrowth above the control level [82]. This lack of effect of NGF on explants up to P0 in age contrasts with previous studies which showed NGF to stimulate sympathetic neurite outgrowth from dispersed adult rat pelvic ganglion neurons [55,56,58]. However, maturation of the pelvic ganglia continues after P0; particularly changes in the pelvic ganglia neurons due to the actions of sex hormones. Thus, it appears that the major actions of NGF occur post-natally.

Neurotrophin-3 (NT-3) is essential during sympathetic neuron development [83-85], but the role of NT-3 in pelvic ganglion development has received only limited attention [49,82]. We found that NT-3 induces strong sympathetic (TH+) and parasympathetic (non-TH+) fibre outgrowth responses from E14.5 onwards.

### Effect of GDNF family ligands on pelvic plexus development

The glial cell line-derived family of neurotrophic factors (GFLs) are implicated in development, maintenance, and plasticity of parasympathetic neurons [43,86,87]. Various studies have also implicated GFLs in the development or plasticity of sympathetic noradrenergic neurons [35,39,88-90]. In addition, the common GFL co-receptor, Ret, was found to be essential for survival of cholinergic sympathetic neurons in the stellate ganglion [91], and the development and/or maintenance of cholinergic sympathetic innervation of sweat glands are at least partly dependent on GFRα2 signaling [92]. Thus, the GFLs are likely to play key roles in the development of the pelvic plexus, where noradrenergic sympathetic, cholinergic sympathetic and parasympathetic classes of neurons are all present.

In adult rat pelvic ganglia, GFRα1 has previously been found to be expressed by many noradrenergic and cholinergic neurons [50]; however, we did not observe detectable GFRα1 immunostaining on TH+ (ie noradrenergic) neurons after E11.5. This may be because GFRα1 is only expressed by TH+ neurons after birth, or due to an interspecies difference. Despite the absence of detectable GFRα1 immunostaining by TH+ cells, we found that GDNF can stimulate outgrowth from both the TH+ and TH- classes of cells. As proposed above for the effects of GDNF-GFRα1 signaling on TH+ cell migration, some GFRα1+/TH- neurites may grow in response to GDNF, and then upregulate TH and downregulate GFRα1 during the culture period. GDNF also induced large increases in parasympathetic fibre outgrowth at E11.5 and E14.5, but had no significant effect on parasympathetic fibre outgrowth at P0. However, neurturin stimulated outgrowth of these fibres at P0, and GFRα2 was upregulated at P0 on the Hu+/TH- cells. The switch from an early importance of GDNF to a later importance for neurturin is fairly common during development of peripheral ganglia [25,26,93].

Gfrα2-/- and Nrtn-/- mice have defects in a number of parasympathetic ganglia [87,93,94] and the innervation of target organs by the parasympathetic component of the pelvic ganglia has also been shown to be altered. A recent study of P0-P21 Nrtn-/- mice has shown that the parasympathetic fibre deficit in each organ is quite variable and can be due to failure of initial projection to the tissue and/or maintenance of the terminal field [3]. Our results also support a role for neurturin in stimulating parasympathetic (non-TH+) fibre outgrowth, as previously shown in adult rat primary neuronal culture [53], because we observed significant increases in non-TH+ fibre outgrowth at E11.5, E14.5 and P0. We also observed an effect of neurturin on sympathetic (ie TH+) fibre outgrowth, although this only occurred at E14.5. However, no major deficits have been identified in noradrenergic sympathetic neurons in Gfrα2-/- and Nrtn-/- mice [3,51,52], so the *in vivo *significance of this data is unclear. We found widespread GFRα2 immunostaining on all neurons within the developing pelvic ganglia, which is in contrast to previous studies on adult mice and rat where no GFRα2 expression was found on the noradrenergic sympathetic neurons [52].

In the adult rat, most noradrenergic neurons, as well as half the penile projecting S1 DRG neurons, contained mRNA for GFRα3 [50]. We could not find detectable levels of GFRα3 protein in pelvic neurons using immunohistochemistry in E11.5 or P0 mice, and levels only barely above background in E14.5 mice. GFRα3 may be upregulated at post-natal stages, especially if it plays a role in the innervation of reproductive tissues, which largely occurs post-natally [3]. Nonetheless, we observed artemin-induced TH+ fibre outgrowth at both E11.5 and P0, but not E14.5. This may be because the number of GFRα3 receptors required to mediate a biological response is below the detection threshold for immunohistochemical detection.

### Neurotrophic factor regulation of transmitter phenotype

Neurotrophic factors can be involved in regulating phenotypic characteristics of neurons, including the expression of peptides and enzymes involved in neurotransmitter pathways. Studies in parasympathetic neurons have demonstrated that lack of neurotrophic support can decrease choline acetyltransferase expression and increase neuropeptide Y expression [55,95]. Of particular relevance to the current study are the effects of neurotrophic factors on TH synthesis: NGF has been found to enhance TH synthesis in adult rat major pelvic ganglion cultures [56]; neurturin reduced the upregulation of TH expression in cultured pelvic parasympathetic neurons [53]; and, GDNF has been demonstrated to downregulate expression of TH in dopamine neurons upon overexpression *in vivo *[96], but to upregulate the expression of TH in a neuroblastoma cell line by increasing the activity of the TH gene promoter and stabilizing TH mRNA [97]. In the current study we analysed the effects of neurotrophins on the TH+ cell migration and TH+/TH- fibre outgrowth from developing pelvic plexus, but we cannot rule out the possibility that the analysis may be confounded in part due to an action of the factors on the phenotype, as well as migration and neurite outgrowth.

## Conclusion

Previous studies have shown that adult pelvic ganglion sympathetic and parasympathetic neurons show differential responses to neurotrophic factors. This study showed that TH+ (sympathetic) neurons are present in the pelvic ganglion primordium from E11.5, and that at this developmental stage, pelvic TH+ neurons already show differences from the non-TH neurons in their neurite outgrowth and migration responses to neurotrophic factors. This may result from the fact that some transcription factors that regulate neurotransmitter expression also regulate expression of receptors for neurotrophic factors. For example, in developing sympathetic ganglia, Phox2b is required for both Ret and TH expression [98]. Further studies are required to determine how expression of the different GFRαs, Trks and p75 are developmentally regulated on cells in pelvic ganglia.

## Authors' contributions

ALS carried out the experiments and the analysis, and drafted the manuscript. RBA participated in the design and coordination of the study and helped draft the manuscript. KK generated the TH-GFP mice and provided comments on the manuscript. HMY conceived of the study, participated in the design and immunohistochemical analysis and helped draft the final manuscript. All authors read and approved the final manuscript.
